# Parental alcohol supply in early childhood and adolescent drinking: Evidence from a prospective cohort

**DOI:** 10.1192/j.eurpsy.2026.10175

**Published:** 2026-03-02

**Authors:** Albert J. Ksinan, Pavla Brennan Kearns, Zuzana Mohrová, Zsófia Csajbók, Jana Klánová, Hynek Pikhart, Martin Bobák

**Affiliations:** 1 RECETOX, Faculty of Science, Masaryk University, Brno, Czech Republic; 2 Department of Public Health, Second Faculty of Medicine, Charles University, Prague, Czech Republic; 3 Department of Psychology and Life Sciences, Faculty of Humanities, Charles University, Prague, Czech Republic; 4 Institute of Epidemiology and Health Care, University College London, London, UK

**Keywords:** adolescence, alcohol use, longitudinal study, parental alcohol consumption, parental alcohol supply, structural equation modeling

## Abstract

**Background:**

Parental alcohol supply in early childhood may increase the risk of alcohol use in late adolescence. This study examined its longitudinal impact and the distinct roles of mothers’ and fathers’ drinking.

**Methods:**

We studied 1,891 mother–child pairs from the Czech European Longitudinal Study of Pregnancy and Childhood. Mothers reported parental alcohol supply at ages 3, 5, 7, and 11 years, while adolescent alcohol use was reported by mothers, pediatricians, and youth at ages 18 and 19 years. Structural equation modeling assessed the longitudinal link between early alcohol supply (three classes: none, occasional, and frequent) and adolescent alcohol use, accounting for parental drinking and covariates, including the child’s sex, mother’s education, and family structure.

**Results:**

Alcohol supply began in early childhood, with 14% of children exposed by age 3 and around 20% by age 11. By age 19, one-third of individuals reported frequent alcohol use. Adolescents’ alcohol use was associated with concurrent mothers’, but not fathers’ alcohol use (*β* = .24, *p* < .001). Early alcohol supply predicted higher adolescent use for both occasional (*β* = .14, *p* = .041) and frequent (*β* = .22, *p* = .005) classes. Mothers’ and fathers’ alcohol use at 6 months was associated with frequent alcohol supply, and fathers’ alcohol use was also associated with occasional alcohol supply. Significant indirect effects were found from early parental drinking to adolescent use via these classes.

**Conclusions:**

Public health messaging should emphasize the risks of early alcohol consumption, including its potential harm to the developing brain.

## Introduction

Early initiation of alcohol use is often facilitated by parents, frequently in the form of supervised sipping. While parents may intend to create a safe environment for this introduction (at home and under supervision), such practices may unintentionally normalize alcohol consumption, set the wrong example for the children, and remove barriers to alcohol access. Some parents believe that sipping under supervision has a protective effect against later alcohol misuse [1, 2]. However, early alcohol introduction may be more harmful than commonly assumed. Children exposed to alcohol at a young age are more likely to experience problematic alcohol use later in life, including alcohol use disorders and alcohol-related harm [3–6]. Research suggests that early alcohol sipping was predictive of alcohol-related problems in late adolescence, even after adjusting for sociocultural and other differences [4, 7, 8].

Although many studies have investigated the relationship between sipping at an early age and later alcohol consumption, rarely has any study examined children younger than 10 years old, as confirmed by a recent review [9]. Moreover, many of the previous studies rely only on a single time point. Alcohol supply in early childhood also differs by cultural and national context. The Czech Republic, a country situated in Central and Eastern Europe, has particularly high levels of alcohol consumption and high rates of alcohol use disorders [10, 11]. Countries with highly prevalent alcohol use disorders may have permissive alcohol-related norms, including parents being more liberal when it comes to their young children trying alcohol.

Using multiple time points across childhood, the aim of the current study was to test whether parental alcohol supply to young children is associated with alcohol use in late adolescence. To the best of our knowledge, this is the first study that examined parental supply of alcohol across early childhood to: (1) assess its prevalence at such a young age and (2) to evaluate if early exposure to alcohol is associated with higher alcohol use longitudinally. Finally, (3) we sought to assess whether previously documented associations between parental alcohol supply and increased late adolescent drinking would also be observed in the unique Czech cultural context.

## Methods

### Participants

**Figure 1.**
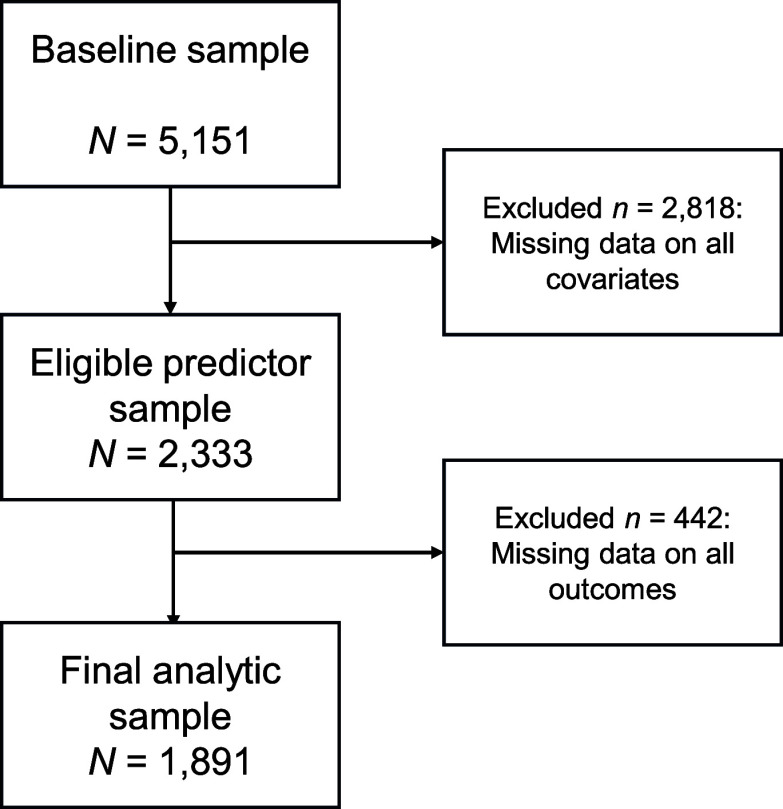
Participant flow diagram in the current study.

The data came from the Czech part of the European Longitudinal Study of Pregnancy and Childhood (ELSPAC-CZ). Pregnant women living in the Brno region or Znojmo district with an expected delivery date between March 1, 1991, and June 30, 1992, were initially enrolled. Information from 5,151 mothers and their newborns was available at baseline [12]. However, given the attrition throughout the study, the analytic sample of the current study included *N* = 1,891 participants (see Figure 1 for participant flow diagram and “Statistical analysis” section for information on handling missing data). The secondary use of all ELSPAC-CZ study data was approved by the (C)ELSPAC Ethics Committee at Masaryk University in Brno, Czech Republic (Ref. No. ELSPAC/EK/1/2014, date 09/17/2014). All participants provided informed consent throughout the study.

The comparison of the baseline sample and the analytical subsample showed that there were no statistically significant differences based on sex, maternal alcohol use, paternal alcohol use, or parental supply of alcohol in the first time point (age 3 years). The mothers in the analytical subsample had higher levels of attained education compared to the full sample (e.g., college-educated 13% vs. 23.9%, *p* < .001). In addition, there was a higher prevalence of two-parent families in the analytical subsample (88% vs. 96%, *p* < .001).

### Measures

#### Exposure: Parental supply of alcohol

The exposure variable was parental supply of alcohol, measured across four time points: 3, 5, 7, and 11 years of the child’s age. At each time point, mothers responded to two questions regarding the child’s alcohol use: “*Has your child ever tasted alcohol?*” and a follow-up, “*If so, how many times in the past month?*” with the option to input any frequency. We have recoded the two questions within each time point into a single item indicator, where 0 = has not tested alcohol, 1 = has tasted once, and 2 = has tasted twice or more times. Then, we defined three classes of children reflecting their history of being supplied alcohol based on the combination of the scores from the four time points. If the children never tasted alcohol during the four time points or tasted only once in one time point (score of 1), they were assigned Class 1 = no or minimal supply of alcohol. If they were supplied with alcohol once in two or three time points or more than once in one time point, they were assigned Class 2 = occasional supply of alcohol. If they were supplied with alcohol in higher quantities, they were assigned to Class 3 = frequent supply of alcohol. If a child had missing data on one or more time points but showed a pattern of drinking sufficient for classification into a higher-risk group (e.g., Class 3) based on the available data, they were still assigned to that class. The classification scheme is shown in Table 1. Based on this classification, 76.9% of children were classified as Class 1, 14.6% as Class 2, and 8.5% as Class 3.

**Table 1.** Coding scheme for parental supply of alcohol across four time points

	Frequency of recorded responses for parental supply of alcohol across four time points		
	0 (has not tasted alcohol)	1 (tasted once during the time point)	2 (tasted more than once during the time point)
Class 1 No or minimal supply of alcohol	4	0	0
	3	1	0
Class 2 Occasional supply of alcohol	3	0	1
	2	2	0
	1	3	0
Class 3 Frequent supply of alcohol	2	1	1
	2	0	2
	1	2	1
	1	1	2
	1	0	3
	0	4	0
	0	3	1
	0	2	2
	0	1	3
	0	0	4

*Note*: The numbers reflect the number of time points (0–4) in each supply category.

#### Outcome: Late adolescence alcohol use

The outcome variable was alcohol use in late adolescence, assessed at ages 18 and 19 years. Given the short time gap between the two assessments and the relatively smaller sample size in each, we combined data from both time points and multiple reporters to maximize the sample size and to construct a single robust indicator of alcohol use at age 18/19 years using a latent factor approach. In this way, we used a total of five indicators of alcohol use at ages 18 and 19 years, using three reporters. At age 18 years, alcohol use was reported by both the adolescent’s pediatrician and mother. Mothers were asked “*Do you think your son/daughter drinks alcoholic beverages?*” with the following response categories: 1 = No, he/she condemns alcohol use; 2 = No he/she is not interested in drinking; 3 = No, but he/she has tried it; 4 = I think he/she occasionally drinks small amounts; 5 = I found out he/she drinks regularly, about once a week; and 6 = Yes, regularly, larger amounts. This was recoded in the following way: 0 (abstainer) = categories 1 and 2; 1 (tried alcohol) = category 3; 2 (regular light/moderate use) = categories 4 and 5; 3 (regular heavy use) = category 6. Pediatricians were asked whether the adolescent consumed alcohol (yes/no). If they answered affirmatively, they rated the frequency and amount of consumption using the following scale: 1 = A glass of wine or a pint of beer not more than once/month; 2 = Larger dose of alcohol not more than once/month; 3 = A glass of wine or a pint of beer more than once/month; 4 = Larger dose of alcohol more than once/month; 5 = A glass of wine or a pint of beer more than once/week; 6 = Larger dose of alcohol more than once/week; 7 = A smaller dose of alcohol daily; and 8 = A larger dose of alcohol daily. We recoded this in the following way: 0 (abstainer) = if they did not drink alcohol; 1 (infrequent use) = categories 1 and 2; 2 (frequent light use) = category 3; 3 (frequent heavy use) = categories 4 through 8. At age 19 years, both pediatricians and mothers completed the same alcohol use items as at age 18 years, which were recoded using the same scheme. In addition, the participants themselves self-reported their alcohol use at age 19 years by responding to the question: “*How many times have you tried alcohol?*,” with the following options: 1 = never; 2 = just once in a lifetime; 3 = more than once in a lifetime; 4 = I use it often.

#### Covariates

We selected the following covariates based on relevant literature: child’s sex, mother’s education, family structure, mother’s alcohol use, and father’s alcohol use. Sex of the child was assessed at birth and was reported by mothers, coded as 0 = male, 1 = female. Mother’s education was assessed in the prenatal period as the highest attained education and was coded in years of education completed, ranging from 8 (elementary school) to 18 years (PhD degree). Family structure was assessed at the age of 3 years for the child. This was coded as 1 = two adults living together, whereas other family structures were coded as 0. Mother’s alcohol use was considered at 6 months and 19 years. At 6 months of the child’s age, mothers reported on their alcohol use by responding to the question: “*How many days in the past month did you drink an amount equivalent to two beers, or 0.5 liters of wine, or four shots of liquor?*” with the following options: 1 = Never; 2 = 1–2 days; 3 = 3–4 days; 4 = 5–10 days; 5 = More than 10 days; 6 = Every day. When children were 19 years old, mothers reported on the number of drinks they had in the past week for each of the following types of alcohol: beer, wine, cocktails, and liquors. We summed up these options and then recoded the number of drinks into the following categories: 1 = 0 drinks; 2 = 1 drink; 3 = 2 to 6 drinks; 4 = 7 to 14 drinks; 5 = 15 or more drinks per week. Similarly, father’s alcohol use was also considered at 6 months and 19 years of the child using identical wordings, which we recoded respectively.

### Statistical analysis

**Figure 2.**
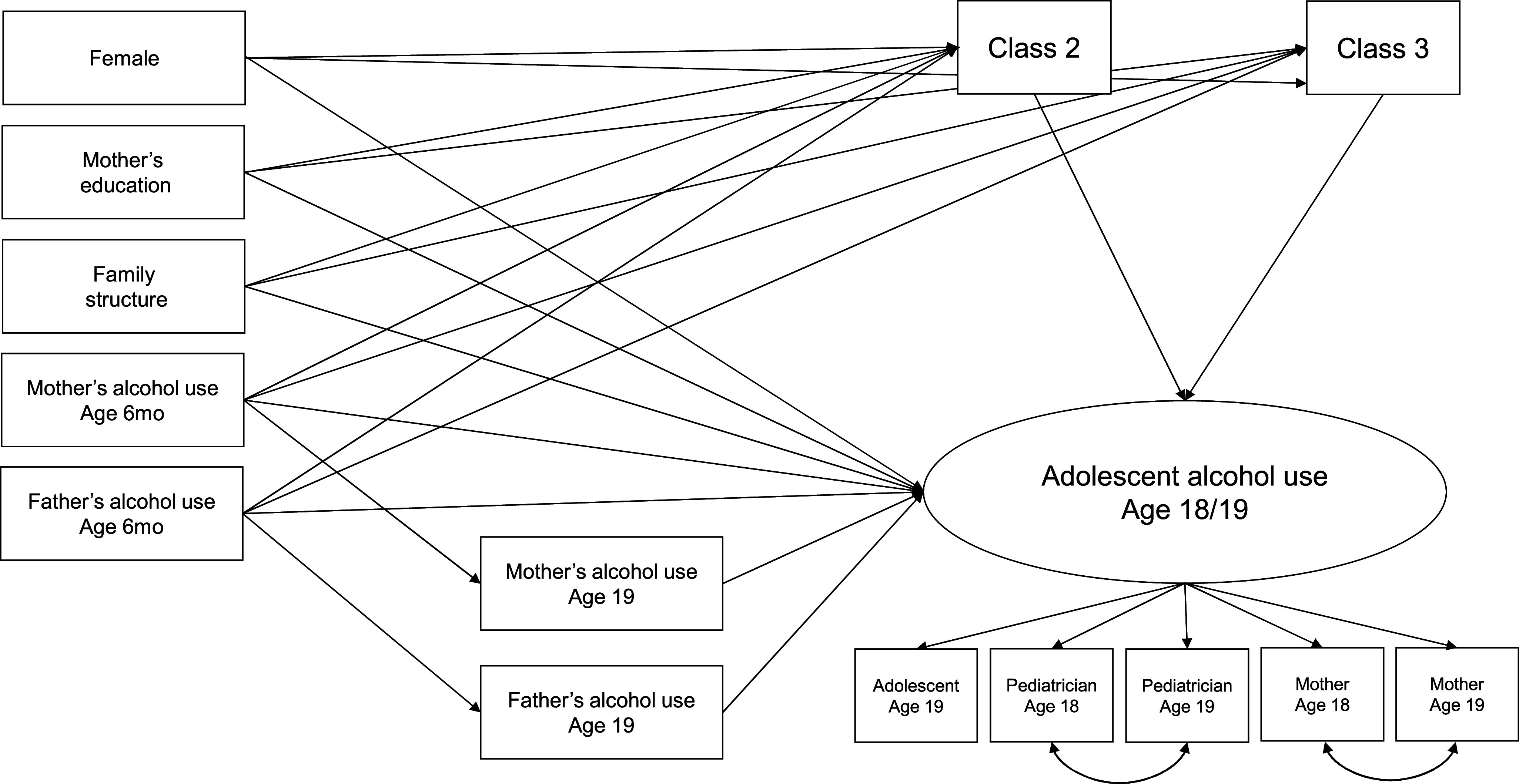
Scheme of the estimated model. *Note*: Class 2 = occasional supply of alcohol, Class 3 = frequent supply of alcohol (Class 1 = reference group).

First, descriptive statistics of study variables were computed. Then, the main research question was tested using structural equation modeling. The outcome (late adolescent alcohol use) was modeled as a latent factor with the five reports (pediatrician age 18 and 19 years, mother 18 and 19 years, and adolescent 19 years) as indicators. The two pediatrician reports and maternal reports were allowed to covary. The effect of alcohol supply classes on the outcome was modeled by creating dummy variables for Classes 2 and 3 (with Class 1 as the reference group). The outcome was regressed on Classes 2 and 3, past and concurrent mothers’ and fathers’ alcohol use (age 19 years), child’s sex, mother’s education, and family structure. To evaluate if more alcohol supply is provided in families where parents drink more, Classes 2 and 3 were regressed on mothers’ and fathers’ alcohol use from the preceding time point (6 months) and covariates. Mothers’ and fathers’ alcohol use at age 19 years was regressed on mothers’ and fathers’ alcohol use at age 6 months. We also tested whether the association between parental drinking and late adolescent alcohol use was mediated by being exposed to alcohol at an early age; that is, classes were mediators of the association between parental alcohol use at 6 months and participants’ alcohol use at age 18/19 years. The estimated model is shown in Figure 2. The significance of the indirect effects was evaluated using 5,000 bootstrapped resamples with bias-corrected confidence intervals (BcCI). All models were estimated in Mplus 8.4 [13]. Analyses were conducted using the weighted least squares (WLSMV) estimator with a probit link function. This approach is appropriate for modeling categorical outcomes and accounts for non-normality in the data. To address missing data, Mplus implements a robust two-stage estimation approach within the WLSMV framework, which maximizes the available data and utilizes information from *N* = 1,891 participants who had at least one value on the covariate and one of the outcomes. The adequacy of model fit was assessed using additional fit index thresholds, recommended by Hu and Bentler (i.e., Comparative Fit Index [CFI] ≥ .95, Root Mean Square Error of Approximation [RMSEA] < .08) [14]. To facilitate public health interpretation, the unstandardized probit coefficients for the focal predictors (supply classes) were converted to approximate odds ratios (ORs). This involved a standard two-step transformation: the probit coefficient is first multiplied by a scaling factor of 1.6 to approximate the logistic regression coefficient (log-odds), which was then exponentiated to obtain the approximate OR [15].

As sensitivity analysis, the full structural model was estimated employing an alternative operationalization of the supply classes: Class 1 = no use (responded with 0 in all four time points; 56.3% of respondents), Class 2 = single time point use (responded with “tasted once” in 1 out of 4 time points; 13.6%), Class 3 = multiple light use (responded with “tasted once” at multiple time points; 11.6%), and Class 4 = heavy use (responded with “tasted twice or more times” in one or more time points; 18.5%). Furthermore, we have decided to use a more stringent criterion for missing values and only include those participants in Classes 1 and 2 that had valid reports for all four time points of data. Class 1 was used as the reference group. Given the more stringent inclusion criteria for defining the parental supply classes, the analytic sample for sensitivity analysis was *N* = 1,513.

## Results

The descriptive statistics of the study variables are shown in Table 2. The parental supply of alcohol was relatively high starting from a very young age, with 9.5% of children having been supplied alcohol once at 3 years of age and 4.6% been supplied more than once at this time point. In total, ~14% of children were supplied alcohol at this time point. This was similar across ages 5 and 7 years. However, at age 11 years, there were about 20% of children who were supplied alcohol (15.6% once and 4.8% more than once).

**Table 2.** Descriptive statistics of the study variables

	*n*	Valid (%)
Parental supply of alcohol (age 3 years)		
Never	2,902	85.9
Once	321	9.5
More than once	157	4.6
Missing	1,771	
Parental supply of alcohol (age 5 years)		
Never	2,463	86.1
Once	282	9.9
More than once	114	4.0
Missing	2,292	
Parental supply of alcohol (age 7 years)		
Never	2,639	86.5
Once	310	10.2
More than once	102	3.3
Missing	2,100	
Parental supply of alcohol (age 11 years)		
Never	1,886	79.6
Once	370	15.6
More than once	113	4.8
Missing	2,782	
Sex		
Female	2,493	51.6
Male	2,655	48.4
Missing	3	
Family structure		
Single-parent family	258	7.5
Two-parent family	3,179	92.5
Missing	1,714	
Mother’s education		
Less than high school	282	7.1
Vocational school	1,247	31.6
High school	1,501	38.0
Some college	199	5.0
College degree	613	15.5
Doctorate	109	2.8
Missing	1,200	
Late adolescent’s report of alcohol use (age 19 years)		
Never	15	4.6
I used it only once	10	3.1%
I used it more than once	194	59.5
I use it often	107	32.8
Missing	4,825	
Mother’s report of adolescent’s alcohol use (age 18 years)		
Abstainer	141	11.1
Tried alcohol before	272	21.3
Regular light/moderate use	860	67.4
Regular heavy use	3	0.2
Missing	3,875	
Mother’s report of adolescent’s alcohol use (age 19 years)		
Abstainer	106	11.5
Tried alcohol before	160	17.3
Regular light/moderate use	648	70.2
Regular heavy use	9	1.0
Missing	4,228	
Pediatrician’s report of adolescent’s alcohol use (age 18 years)		
Abstainer	39	7.6
Infrequent use	150	29.2
Frequent light use	40	7.8
Frequent heavy use	284	55.4
Missing	4,638	
Pediatrician’s report of adolescent’s alcohol use (age 19 years)		
Abstainer	21	8.2
Infrequent use	50	19.6
Frequent light use	17	6.7
Frequent heavy use	167	65.5
Missing	4,896	
Mother’s alcohol use (age 6 months)		
Never	2,930	68.4
1–2 days	933	21.8
3–4 days	199	4.6
5–10 days	95	2.2
More than 10 days	122	2.8
Every day	2	0.0
Missing	870	
Mother’s alcohol use (age 19 years)		
No drinks	277	29.6
1 drink a week	107	11.4
2–6 drinks a week	438	46.8
7–14 drinks a week	101	10.8
More than 15 drinks a week	13	1.4
Missing	4,215	
Father’s alcohol use (age 6 months)		
Never	838	21.9
1–2 days	1,162	30.3
3–4 days	791	20.6
5–10 days	585	15.3
More than 10 days	363	9.5
Every day	96	2.5
Missing	1,316	
Father’s alcohol use (age 19 years)		
No drinks	101	15.2
1 drink a week	32	4.8
2–6 drinks a week	249	37.4
7–14 drinks a week	193	29.0
More than 15 drinks a week	90	13.5
Missing	4,486	

About one-third of 19-year-olds reported that they used alcohol often, while <5% said they never used it. Regarding maternal reports, about 11% of mothers at ages 18 and 19 years reported that their children did not use alcohol, while about 70% reported that their children used it regularly, but not heavily. Pediatrician reports showed that about 8% of adolescents aged 18 and 19 years were abstainers, while about 55% reported frequent heavy use at age 18 years and 66% at age 19 years. While more than two-thirds of mothers at 6 months of the child’s age reported no drinking, only about 22% of fathers at the same time point reported the same. At age 19 years, more than 40% of fathers and about 12% of mothers reported drinking one or more drinks a week.

**Figure 3.**
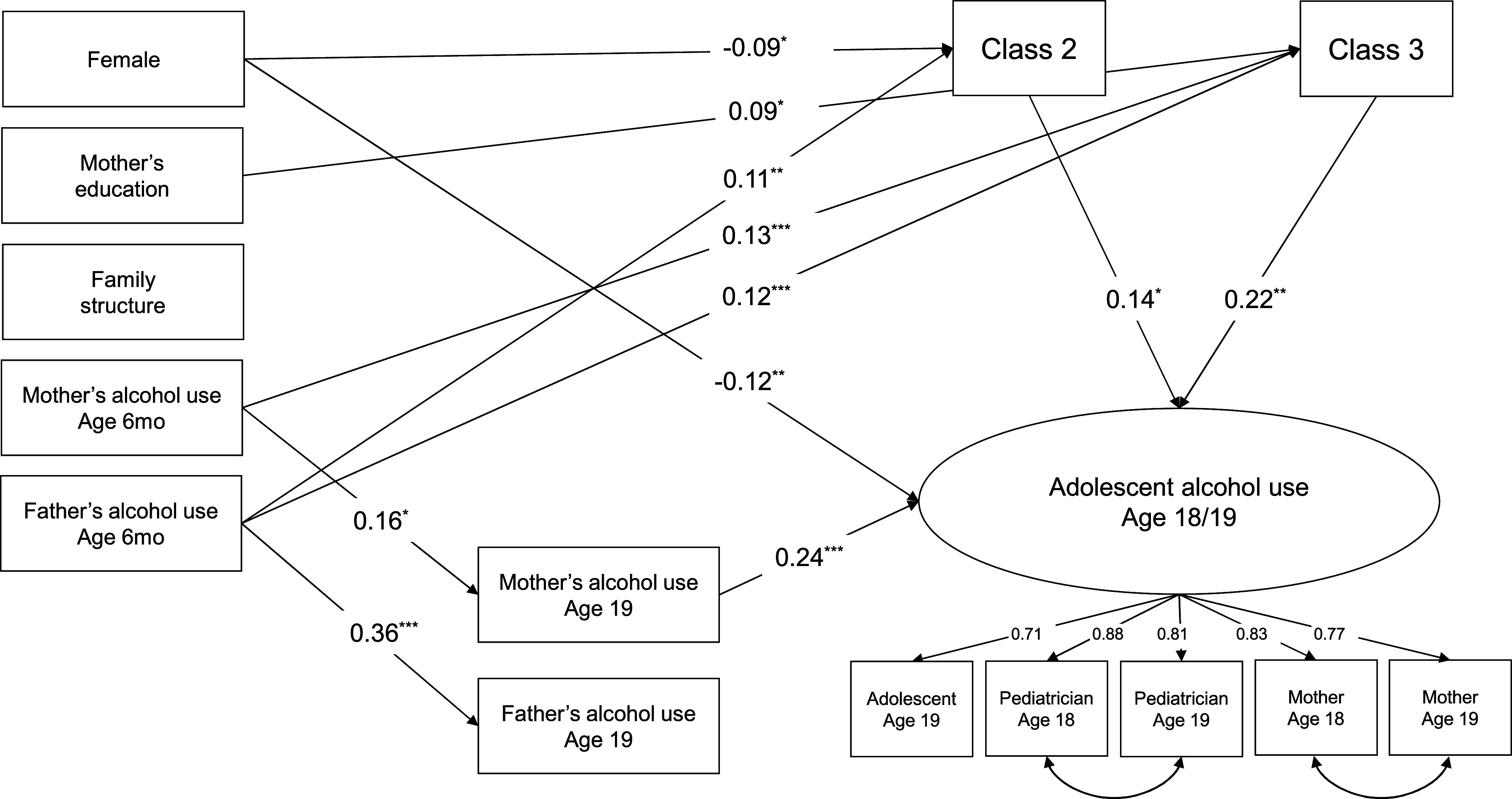
Results from the full model are estimated. *Note*: Standardized estimates. Only paths statistically significant at *p* < .05 are shown. All factor loadings are significant at *p* < .001. *p < .05, **p < .01, ***p < .001.

In the next step, the full structural model was estimated. The full model showed an adequate fit, *χ* ^2^(54) = 102.79, *p* < .001, CFI = .97, RMSEA = .02, 90% CI = [.02, .03]. Standardized model results are shown in Figure 3. All five indicators had substantial loadings on the latent factor (all *λ* ≥ 0.71), suggesting a good overlap in the construct. Girls showed lower rates of alcohol use as compared to boys, standardized *β* = −.12, *p* = .009. Adolescents’ alcohol use was associated with concurrent mother’s alcohol use, *β* = .24, *p* < .001, but not with father’s. Importantly, individuals belonging to Class 2 (occasional supply: *β* = .14, *p* = .041) or Class 3 (frequent supply: *β* = .22, *p* = .005) had higher rates of alcohol use, as compared to Class 1 (no supply). Converting the latent unstandardized probit coefficients to approximate ORs, the membership in Class 2 was associated with 1.21 times higher odds of increasing alcohol use (95% CI = [1.01, 1.45]), while for Class 3, the odds increased to 1.33 (95% CI = [1.10, 1.61]).

Higher father’s alcohol use at 6 months was associated with a higher likelihood of belonging to Class 2 (*β* = .11, *p* = .003) as well as Class 3 (*β* = .12, *p* = .002). In addition, mother’s alcohol use at 6 months was associated with Class 3 (*β* = .13, *p* < .001). Mother’s (*β* = .16, *p* = .002) and father’s (*β* = .36, *p* < .001) alcohol use showed significant time stability across 19 years. Significant indirect effect was found from mother’s alcohol use at the child’s age of 6 months to late adolescent alcohol use through Class 3 (*B* = 0.026, 95% BcCI = [.007, .056]) and from father’s alcohol use at 6 months through Class 2 (*B* = 0.009 [.001, .025]) as well as Class 3 (*B* = 0.017 [.004, .037]). The full model explained 18.7% of the variance in late adolescent alcohol use.

### Sensitivity analysis

The results using alternative class distinction mirror the findings from the main analyses. Specifically, there was an increase in the association between the frequency of parental supply and later alcohol use: Class 2 (*β* = .15, *p* = .021), Class 3 (*β* = .22, *p* = .020), and Class 4 (*β* = .24, *p* < .001; all compared to Class 1 – no supply). No significant indirect effect was found for Class 2 or Class 3. However, for Class 4, a significant indirect effect on late adolescent alcohol use was found for both paternal alcohol use (*B* = 0.036 [.014, .069]), as well as maternal alcohol use (*B* = 0.025 [.007, .052]) at the child’s age of 6 months.

## Discussion

The present study, capitalizing on a prospective longitudinal cohort from the Czech Republic, found that about 14% of children at ages of 3, 5, and 7 years got to taste alcohol at least once, while this rate increased to 20% at the age of 11 years. Our main analysis suggests that patterns of early alcohol supply from parents, operationalized as three classes, were meaningfully linked to the children’s alcohol use in late adolescence. Specifically, the participants who experienced frequent or occasional supply of alcohol grew up to use alcohol more heavily at ages 18–19 years compared to those who never received alcohol from their parents. Mother’s concurrent alcohol use also contributed to adolescent drinking, while father’s current drinking did not have the same effect. Girls tended to use alcohol less than boys. Early parental drinking – both from mothers and fathers when the child was an infant – significantly increased the likelihood of being supplied alcohol by parents, and this pathway carried through to late adolescent alcohol use. Overall, both early parental drinking and later parental supply contributed to a chain of risk that culminated in greater alcohol use in late adolescence.

There is limited research on alcohol use in children under the age of 10 years, with most existing studies focusing on children aged 10 years and older [9, 16]. A systematic review by Van De Kurk reported that the prevalence of alcohol supply to children ranged from 6 to 60%, depending on the study population and study design [17]. Similarly, a review by Skylstad found a comparable prevalence of alcohol use among children [9]. An Australian study found that the prevalence of parental alcohol supply of 15% at age 13 years [4], while another Australian study observed a decrease in parental alcohol supply between 2004 and 2013 (from 16 to 8%) [18]. In the United States, one study for children at the mean age of 12 years reported a sipping prevalence of 9.8% [19], whereas another found that nearly 30% of children aged 11 years had sipped alcohol [20]. The authors also showed that children who sipped were more likely to drink heavily in a few years’ time [20]. A recent study from the United States looking at children aged 9–13 years reported that their sipping prevalence was 22.5% and significantly higher in males [21]. In the United Kingdom, 15% of 11-year-olds had ever had alcohol [22]. A study conducted in Japan showed that 96.4% of parents with children in elementary school supplied alcohol to their children; however, no age was provided [23]. The discrepancies in reported prevalences across studies could be attributed to many factors. These include differences in data collection, study design, time frames, national and cultural norms regarding alcohol consumption, and differences in sociodemographic conditions, which may influence rates in either direction.

The current study offers several novel contributions. First, it provides a rare longitudinal assessment of alcohol use beginning in early childhood, whereas most previous studies have assessed parental alcohol supply starting around age 10 years and often without repeated measurement. A key and novel finding is the longitudinal positive association between parental alcohol supply in early childhood and alcohol use in late adolescence – even after adjusting for both baseline and concurrent parental alcohol use. Notably, the study was conducted in the Czech Republic, a country characterized by permissive alcohol regulations and high levels of alcohol use [24]. Our findings are consistent with those of Chan et al., who examined the link between alcohol supply and adolescent alcohol use across 45 low- and middle-income countries. They reported that adolescents in countries with higher levels of early alcohol supply were more likely to initiate drinking at an earlier age [25].

Several mechanisms may underlie the observed associations. First, parental consumption normalizes alcohol use in the household, increases its availability, and may structure leisure activities around drinking [26]. Our findings indicate that mothers’ alcohol use had a stronger influence on children’s drinking compared to fathers’ use. This may be due to mothers’ traditional role as primary caregivers, their closer emotional bond with the child, and the fact that mothers’ drinking may signal broader psychosocial difficulties, as alcohol use is generally more normalized among men [27]. We have previously shown that prenatal alcohol exposure from mothers, but not fathers, is associated with poorer mental health and behavioral outcomes in children [28]. This supports the hypothesis that mothers’ drinking may exert both biological and social influences that are stronger than those of fathers’ drinking. Moreover, we have shown that early alcohol supply was associated with increased alcohol use in late adolescence, independent of parental drinking. This suggests a potential neurobiological impact of early alcohol exposure on brain development. For instance, the dorsal anterior cingulate cortex (dACC), involved in cognitive control and response inhibition, moderated the longitudinal effects of early sipping on personality and depression. Bilateral dACC activation during a stop-signal task predicted changes in these outcomes. Furthermore, bidirectional relationships were observed between early sipping and personality traits, where poor baseline inhibition and increased sipping appeared to accelerate shifts in personality and depressive symptoms as children transitioned into adolescence [29].

The major strengths of the current study include its longitudinal design starting in early childhood, a large sample size, and a latent outcome derived from multiple informants. However, several limitations must be noted. First, regarding generalizability, the analytic sample was more highly educated and socially stable than the original cohort. While we adjusted for these factors within the structural model to minimize parameter bias, our findings may not fully generalize to more structurally disadvantaged families. Furthermore, the Moravian region, where the respondents come from, may have a more permissive attitude toward underage drinking compared to other parts of the Czech Republic.

Second, there is a risk of measurement and reporting bias. Maternal and pediatrician reports of adolescent drinking are likely conservative estimates, and the true associations could have been stronger if more precise measures were available. Nonetheless, all outcome indicators loaded adequately onto the latent construct, suggesting they successfully captured the shared underlying concept of alcohol involvement. Finally, participant attrition, a common challenge in longitudinal birth cohorts, may have led to an underestimation of effects. If adolescents with higher alcohol use were more likely to drop out, the true associations between early parental supply and later use could be even stronger than those reported here.

The findings underscore the persistent risk associated with early parental alcohol supply and its long-term impact on adolescent drinking behavior. It emphasizes the need for clearer parental guidelines and early preventive efforts to delay alcohol exposure in childhood. Public health messaging should emphasize the risks of early alcohol consumption, including its potential adverse effects on brain development.

## Data Availability

The data analyzed in this study are available upon request through the website of the Czech ELSPAC project, https://www.celspac.cz/ (accessed on January 22, 2026).
