# Parental alcohol supply in early childhood and adolescent drinking: Evidence from a prospective cohort – ADDENDUM

**DOI:** 10.1192/j.eurpsy.2026.12207

**Published:** 2026-04-16

**Authors:** Albert J. Ksinan, Pavla Brennan Kearns, Zuzana Mohrová, Zsófia Csajbók, Jana Klánová, Hynek Pikhart, Martin Bobák

**Affiliations:** 1 RECETOX, Faculty of Science, Masaryk University, Brno, Czech Republic; 2 Department of Public Health, Second Faculty of Medicine, Charles University, Prague, Czech Republic; 3 Department of Psychology and Life Sciences, Faculty of Humanities, Charles University, Prague, Czech Republic; 4 Institute of Epidemiology and Health Care, University College London, London, UK

The authors regret that this article was originally published with incomplete author affiliations. In addition to their stated affiliations, Martin Bobák and Hynek Pikhart are affiliated with RECETOX, Faculty of Science, Masaryk University. The original article has now been updated.
